# Longitudinal relationships between psychological distress and subsequent cognitive decline and dementia: a multi‐cohort study

**DOI:** 10.1002/alz70860_105566

**Published:** 2025-12-23

**Authors:** Jean Stafford, Serhiy Dekhtyar, Archana Singh‐Manoux, Tom C Russ, Kate Walters, Neil Davies, Vasiliki Orgeta, Jane Maddock, Marcus Richards, James Kirkbride, Robert Howard, Praveetha Patalay

**Affiliations:** ^1^ University of Edinburgh, Edinburgh, United Kingdom; ^2^ Karolinska Institutet, Stockholm, Sweden; ^3^ UCL, London, United Kingdom; ^4^ University College London, London, United Kingdom

## Abstract

**Background:**

Psychological distress has shown links with cognitive functioning in late‐life, although the nature of the association remains unclear. Using a multi‐cohort approach, we examined longitudinal associations of psychological distress with cognition and dementia, testing whether findings varied by severity, persistence and type of psychiatric symptom. The role of temporality, age‐at‐symptom‐assessment, sex, and socio‐economic position will also be examined.

**Method:**

We used five longitudinal studies: Caerphilly Prospective Study (CAPS), English Longitudinal Study of Ageing (ELSA), National Child Development Study (NCDS), National Survey of Health and Development (NSHD), and Whitehall II (WHII). Psychological distress and global cognition were standardised to facilitate cross‐study and longitudinal comparisons. Associations with cognition were examined using linear regression and mixed effects models, with Cox and logistic regression models applied to examine links with dementia. Results were pooled using two‐stage individual participant data meta‐analysis.

**Results:**

Pooled analyses (total *N* = 20,934; five studies) showed greater psychological distress was associated with poorer global cognitive score (β=‐0.04 [95%CI: ‐0.07; ‐0.01]; I2=74.6%), including with cut‐offs indicating clinically‐significant distress (β=‐0.08 [‐0.15; ‐0.01]; I2=70.6%). Associations were present for both persistent (β=‐0.16 [‐0.27; ‐0.06]; I2=78.1%) and intermittent distress (β=‐0.09 [‐0.12; ‐0.05]; I2=79.1%), and were observed individually for depressive and anxiety subscales. Baseline distress was not associated with cognitive decline pooling across three studies. In ELSA, persistent (β=‐0.008 [‐0.016; ‐0.002]), but not intermittent distress was associated with cognitive decline.

Psychological distress was associated with subsequent dementia in ELSA (hazard ratio (HR)=1.11 [1.00; 1.22]) and CAPS (odds ratio (OR)=1.29 [1.07; 1.57]), including for clinically‐significant symptoms (ELSA HR=1.29 [1.02; 1.63]; CAPS OR=1.64 [1.05; 2.56]). In WHII, associations with subsequent dementia were not found with baseline psychological distress (1985‐1988), but became apparent when distress was assessed later (2001 HR=1.27 [1.06; 1.52]).

**Conclusions:**

In this multi‐cohort study, psychological distress was associated with subsequent dementia and poorer cognitive scores, although between‐study heterogeneity was high. Associations were present for both depressive and anxiety symptoms, for clinically‐significant symptoms, and were stronger for persistent distress. Findings highlight the potential relevance of psychological distress in informing prevention and early detection approaches in dementia, both of which are major global public health priority areas.

